# Rehabilitation following rotator cuff repair: A work of the Commission Rehabilitation of the German Society of Shoulder and Elbow Surgery e. V. (DVSE) in collaboration with the German Association for Physiotherapy (ZVK) e. V., the Association Physical Therapy, Association for Physical Professions (VPT) e. V. and the Section Rehabilitation—Physical Therapy of the German Society for Orthopaedics and Trauma e. V. (DGOU)

**DOI:** 10.1007/s11678-018-0448-2

**Published:** 2018-02-22

**Authors:** Christian Jung, Lena Tepohl, Reina Tholen, Knut Beitzel, Stefan Buchmann, Thomas Gottfried, Casper Grim, Bettina Mauch, Gert Krischak, Hans Ortmann, Christian Schoch, Frieder Mauch

**Affiliations:** 1 Schulthess Klinik Zürich, Obere Extremitäten, Lengghalde 2, 8008 Zürich, Switzerland; 2 Institut für Rehabilitationsmedizinische Forschung an der Universität Ulm, Bad Buchau, Germany; 3 Deutscher Verband für Physiotherapie (ZVK) e. V., Köln, Germany; 4 Abteilung und Poliklinik für Sportorthopädie, Klinikum rechts der Isar, München, Germany; 5 Orthopädisches Fachzentrum (OFZ), Weilheim i. Obb., Germany; 6 Klinik Hoehenried gGmbH der Deutschen Rentenversicherung Bayern Sued, Bernried, Germany; 7 Klinikum Osnabrück GmbH, Osnabrück, Germany; 8 Klinikum Stuttgart—Bad Cannstatt, Stuttgart, Germany; 9 Federseeklinik Bad Buchau, Bad Buchau, Germany; 10 Verband Physikalische Therapie (VPT) e. V. Landesgruppe Bayern, München, Germany; 11 St. Vinzenz Allgäu, Pfronten, Germany; 12 Sportklinik Stuttgart, Stuttgart, Germany; 13 Kommission Rehabilitation der Deutschen Vereinigung für Schulter und Ellenbogenchirurgie e. V. (DVSE), Stuttgart, Germany; 14 Sektion Rehabilitation – Physikalische Therapie der Deutschen Gesellschaft fuer Orthopaedie und Unfallchirurgie e. V. (DGOU), Berlin, Germany

**Keywords:** Treatment outcome, Rotator cuff repair, Tendon reconstruction, Cuff tear, Physiotherapy

## Abstract

**Background:**

Tears and lesions of the rotator cuff are a frequent cause of shoulder pain and disability. Surgical repair of the rotator cuff is a valuable procedure to improve shoulder function and decrease pain. However, there is no consensus concerning the rehabilitation protocol following surgery.

**Objectives:**

To review and evaluate current rehabilitation contents and protocols after rotator cuff repair by reviewing the existing scientific literature and providing an overview of the clinical practice of selected German Society of Shoulder and Elbow Surgery e. V. (DVSE) shoulder experts.

**Materials and methods:**

A literature search for the years 2004–2014 was conducted in relevant databases and bibliographies including the Guidelines International Network, National Guidelines, PubMed, Cochrane Central

Register of Controlled Trials, Cochrane Database of Systematic Reviews, and the Physiotherapy Evidence Database. In addition, 63 DVSE experts were contacted via online questionnaire.

**Results:**

A total of 17 studies, four reviews and one guideline fulfilled the inclusion criteria. Based on these results and the obtained expert opinions, a four-phase rehabilitation protocol could be developed.

## Introduction

Tears of the rotator cuff tendons (RC) are a frequent cause of shoulder complaints [44]. Improvement in terms of strength, movement and pain reduction can be expected after rotator cuff repair surgery [27]. Unfortunately, there is no consensus on the rehabilitation protocols and contents following the surgical procedure [24]. Conventional rehabilitation protocols after reconstruction of the rotator cuff (RCR) often vary considerably, even in terms of basic content such as the length of immobilization, movement limitations and whether or not an orthosis should be used. There still is a lack of evidence for many common forms of rehabilitation contents, although in many health care systems evidence-based medicine has gained ground. In Germany, among others, the guideline program of the German Pension Insurance Association focused on this conflict [23, 25].

The rehabilitation commission of the German Society for Shoulder and Elbow Surgery (DVSE) has studied this issue intensively. The aim of this paper was, firstly, to conduct an evidence-based evaluation of the most important forms of treatment after RCR, based on an extensive literature review and, with the help of a survey among DVSE shoulder experts, to determine if there is an existing best-clinical practice consensus for or against specific forms of treatment.

## Materials and methods

### Literature review

**Fig. 1:**
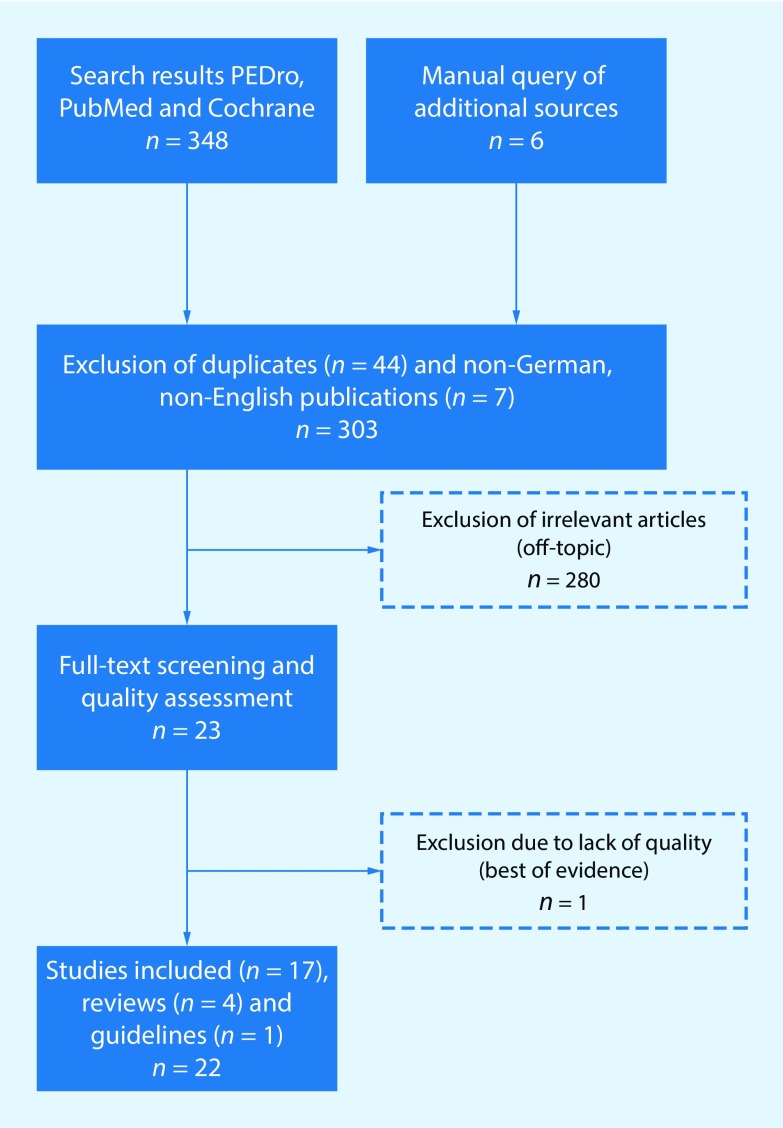
Systematic review search algorithm

The literature search had a hierarchical structure (best available evidence) based on guidelines, health technology assessments (HTA), systematic reviews and clinical studies that investigated post-operative rehabilitation after RCR. This was supplemented by an analysis of the primary literature under examination (Fig. 1). We started by searching for national and international guidelines in the databases of the “Guidelines International Network” (http://www.g-i-n.net/), various other national guidelines (National Guideline Clearinghouse, AWMF, SIGN, NICE) and HTA (INAHTA, HTAi, EUnetHTA, DIMDI, IQWiG).

A search for meta analyses, systematic reviews and primary studies was conducted using the electronic databases Medline via PubMed, the Cochrane Central Register of Controlled Trials, the Cochrane Database of Systematic Reviews and the Physiotherapy Evidence Database (PEDro). The period between 1/2004 and 10/2014 was the period of reference. In addition, a manual search was conducted that included general internet research, a screening of the literature references listed in the collected articles, and a renewed assessment of various journals. A manual search for relevant animal studies was also performed for the topics of tendon healing and length of immobilization. The relevant publications were selected based on how relevant their content was to the issue, whether they were in English or German, and whether the comparative studies enlisted at least ten patients per group.

The literature was selected based on the PICO concept of the Cochrane Institute (Table 1). The levels of evidence were interpreted based on the classifications of the Oxford Center for Evidence-Based Medicine 2009 (OCEBM).

**Table 1:** PICO-System (Cochrane Institute)

P	Population	Patients in post operative rehabilitation after rotator cuff repair
I	Intervention	Specific treatment modalities during post operative rehabilitation after rotator cuff repair (e. g. frequency, duration and interval of therapy)
C	Comparison	Patients without specific treatment modalities during post operative rehabilitation after rotator cuff repair
O	Outcome	Impact of treatment modalities during post operative rehabilitation after rotator cuff repair (e. g. improvement of function, pain or quality of life)

The PEDro scale (http://www.pedro.org.au) was used to analyze the individual studies and the systematic reviews were assessed according to AMSTAR (assessment of multiple systematic reviews; http://amstar.ca). Only studies that demonstrated the highest obtainable level of evidence served as the basis for the consensus paper. At least two studies were required per topic. For example, if there was only one Level I study done on the topic, Level II studies were also taken into account. If there were two or more Level I studies, no Level II, III and IV studies were considered. Consensus topics that were not presented accordingly in the literature were included on the basis of “best available evidence”.

### Expert opinions

After evaluating the literature, the DVSE’s Rehabilitation Commission decided which topics required the opinions of the DVSE experts. The individual topics were assigned to the following groups:

1. Immobilization and arm positioning
2. Physical therapy (cryotherapy, electrotherapy, hydrotherapy)
3. Physiotherapy, self-exercise and CPM (continuous passive motion)
4. Rehabilitation protocols

The online tool Surveymonkey (www.surveymonkey.com) was used to survey 63 selected DVSE experts between 2/2015 and 4/2015. The participation rate was 69.8%.

## Results and discussion

The guideline search resulted in one hit for the American Academy of Orthopedic Surgeons. Two systematic reviews and 13 clinical studies in the databases were examined. A manual search revealed two additional systematic reviews and four individual studies. An overview of all the papers is listed in Tables 2, 3 and 4.

**Table 2:** Search results reviews

Author	Year	Title	Level of Evidence	Studies	Outcome-Measure	Result/Conclusion
Chan et al	2014	Delayed versus early motion after arthroscopic rotator cuff repair: a meta-analysis	Review 1A/1+	3	Primary outcome: functional scores from the validated ASES scale Secondary outcome: Constant-Murley scale (CMS), Simple Shoulder Test (SST), Western Ontario Rotator Cuff (WORC) index, and Disabilities of the Arm, Shoulder, and Hand (DASH)	Three level I and 1 level II randomized trials were eligible and included. Pooled analysis revealed no statistically significant differences in American Shoulder and Elbow Surgeons scores between delayed vs early motion rehabilitation (mean difference [MD], 1.4; 95% confidence interval [CI], −1.8 to 4.7; *P* = 0.38, I(2) = 34%). The risk of retears after surgery did not differ statistically between treatment groups (risk ratio, 1.01; 95% CI, 0.63–1.64; *P* = 0.95). Early passive motion led to a statistically significant, although clinically unimportant, improvement in forward elevation between groups (MD, −1°; 95% CI, −2° to 0°; *P* = 0.04, I(2) = 0%). There was no difference in external rotation between treatment groups (MD, 1°; 95% CI, −2° to 4°; *P* = 0.63, I(2) = 0%). None of the included studies identified any cases of postoperative shoulder stiffness
Shen et al	2014	Does immobilization after arthroscopic rotator cuff repair increase tendon healing? A systematic review and meta-analysis	Review 1A/1+	3	Primary outcome: tendon healing in the repaired cuff Secondary outcome: range of motion (ROM) and American Shoulder and Elbow Surgeons (ASES) shoulder scale, Simple Shoulder Test (SST), Constant, and visual analog scale (VAS) for pain scores	Three randomized controlled trials (RCTs) examining 265 patients were included. Meta-analysis revealed no significant difference in tendon healing in the repaired cuff between the early-motion and immobilization groups. A significant difference in external rotation at 6 months postoperatively favored early motion over immobilization, but no significant difference was observed at 1 year postoperatively. In one study, Constant scores were slightly higher in the early-motion group than in the immobilization group. Two studies found no significant difference in ASES, SST, or VAS score between groups
Du Plessis et al	2011	The effectiveness of continuous passive motion on range of motion, pain and muscle strength following rotator cuff repair: a systematic review	Review 1A/1++	3	Shoulder joint range of motion as measured by a goniometer, shoulder score and the constant score; shoulder pain as measured by the visual analogue scale and the shoulder score; and shoulder muscle strength as measured by the hand-held dynamometer and the shoulder score	Continuous passive motion is safe to use with physiotherapy treatment following rotator cuff repair surgery. It may help to prevent secondary complications post operatively
Baumgarten et al	2009	Rotator cuff repair rehabilitation: a level I and II systematic review	Review 1A/1+	4	Hospital for Special Surgery System for Assessing Shoulder Function, Mayo Clinic preoperative and postoperative analysis of the shoulder, pain VAS, range of motion, isometric strength, Shoulder Pain and Disability Index (SPADI), Shoulder Service Questionnaire (modified version of the Shoulder Rating Questionnaire)	Two studies examined the use of continuous passive motion for rotator cuff rehabilitation, and 2 studies compared an unsupervised, standardized rehabilitation program to a supervised, individualized rehabilitation program. These studies did not support the use of continuous passive motion in rotator cuff rehabilitation, and no advantage was shown with a supervised, individualized rehabilitation protocol compared to an unsupervised, standardized home program. Each investigation had weaknesses in study design that decreased the validity of its findings

**Table 3:** Search results guidelines

Editor	Year	Title	Recommendation & Statement
American Academy of Orthopaedic Surgeons (AAOS)	2010	Optimizing the Management of Rotator Cuff Problems—Guideline and Evidence Report	**Post-Operative Treatment—Cold Therapy** In the absence of reliable evidence, it is the opinion of the work group that local cold therapy is Beneficial to relieve pain after rotator cuff surgery. Strength of Recommendation: Consensus
			**Post-Operative—Sling, shoulder immobilizer, abduction pillow, or abduction brace** We cannot recommend for or against the preferential use of an abduction pillow versus a standard sling after rotator cuff repair. Strength of Recommendation: Inconclusive
			**Post-Operative Rehabilitation—Range of Motion Exercises** We cannot recommend for or against a specific time frame of shoulder immobilization without range of motion exercises after rotator cuff repair. Strength of Recommendation: Inconclusive
			**Post-Operative Rehabilitation—Active Resistance Exercises** We cannot recommend for or against a specific time interval prior to initiation of active resistance exercises after rotator cuff repair. Strength of Recommendation: Inconclusive
			**Post-Operative Rehabilitation—Home Based Exercise and Facility Based Rehabilitation** We cannot recommend for or against home-based exercise programs versus facility-based rehabilitation after rotator cuff surgery. Strength of Recommendation: Inconclusive
			**Post-Operative—Infusion Catheters** We cannot recommend for or against the use of an indwelling subacromial infusion catheter for pain management after rotator cuff repair. Strength of Recommendation: Inconclusive

**Table 4:** Search results original studies

Author	Year	Title	Level of Evidence	No. of patients (*n* =)	Outcome-Measure	Result
Arndt et al	2012	Immediate passive motion versus immobilization after endoscopic supraspinatus tendon repair: a prospective randomized study	RCT 2b/1−	100 50/50	Passive range of motion with a goniometer (in anterior elevation and external rotation) and Constant and Murley score	The mean preoperative Constant score improved significantly from 46.1 points to 73.9 at the final follow-up. The rate of intact cuffs was 58.5%. Functional results were statistically better after immediate passive motion with a mean passive external rotation of 58.7° at the final follow-up versus 49.1° after immobilization (*P* = 0.011), a passive anterior elevation of 172.4° versus 163.3° (*P* = 0.094) respectively, a Constant score of 77.6 points versus 69.7 (*P* = 0.045) respectively, and a lower rate of adhesive capsulitis and complex regional pain syndrome. Results for healing seemed to be slightly better with immobilization, but this was not statistically significant: the cuff had a normal appearance in 35.9% of cases after immobilization compared to 25.6% after passive motion, an image of intratendinous addition was found in 25.6% versus 30.2%, punctiform leaks in 23.1% versus 20.9%, and recurrent tears in 15.4% versus 23.3% respectively
Blum et al	2009	Repetitive H‑wave device stimulation and program induces significant increases in the range of motion of post operative rotator cuff reconstruction in a double-blinded randomized placebo controlled human study	RCT 2b/1−	22 12/10	Range of motion	Patients who received HWDS compared to PLACEBO demonstrated, on average, significantly improved range of motion. Results confirm a significant difference for external rotation at 45 and 90 days postoperatively; active range at 45 days postoperatively (*p* = 0.007), active at 90 days postoperatively (*p* = 0.007). Internal rotation also demonstrated significant improvement compared to PLACEBO at 45 and 90 days postoperatively; active range at 45 days postoperatively (*p* = 0.007), and active range at 90 days postoperatively (*p* = 0.006). There was no significant difference between the two groups for strength testing
Brady et al	2008	The addition of aquatic therapy to rehabilitation following surgical rotator cuff repair: a feasibility study	RCT 2b/1−	18 12/6	Passive range of motion; Ontario Rotator Cuff Index	There was a significant improvement in both range of motion and Western Ontario Rotator Cuff scores in all subjects with treatment (*p* < 0.001). Furthermore, participation in aquatic therapy significantly improved passive flexion range of motion measures at three weeks (mean 46°, 95% CI 17–75, *p* = 0.005) and six weeks (30°, 95% CI 8–51, *p* = 0.01). There was no significant difference in the attendance rates (80% in both groups) or patients perceptions of the programmes (100% confidence and assurance in both groups)
Cuff et al	2012	Prospective randomized study of arthroscopic rotator cuff repair using an early versus delayed postoperative physical therapy protocol	RCT 2b/1−	68 33/35	American Shoulder and Elbow (ASES) questionnaire, Simple Shoulder Test (SST) scores and range of motion (digitally recorded)	Both groups had similar improvements in preoperative to postoperative American Shoulder and Elbow Surgeons scores (early group: 43.9 to 91.9, *p* < 0.0001; delayed group: 41.0 to 92.8, *p* < 0.0001) and Simple Shoulder Test scores (early group: 5.5 to 11.1, *p* < 0.0001; delayed group: 5.1 to 11.1, *p* < 0.0001). There were no significant differences in patient satisfaction, rotator cuff healing, or range of motion between the early and delayed groups
Düzgün et al	2011	Comparison of slow and accelerated rehabilitation protocol after arthroscopic rotator cuff repair: pain and functional activity	RCT 2b/1−	29 13/16	Disabilities of The Arm Shoulder and Hand (DASH) questionnaire, active range of motion	There was no significant difference between the slow and accelerated protocols with regard to pain at rest (*p* > 0.05). However, the accelerated protocol was associated with less pain during activity at weeks 5 and 16, and with less pain at night during week 5 (*p* < 0.05). The accelerated protocol was superior to the slow protocol in terms of functional activity level, as determined by DASH at weeks 8, 12, and 16 after surgery (*p* < 0.05)
Ellsworth et al	2006	Electromyography of Selected Shoulder Musculature During Unweighted and Weighted Pendulum Exercises	Case-control study 3a/2−	26 9/17	Muscle activity (EMG)	When grouped across all patients and all other factors included in the ANOVA, the type of pendulum exercise did not have a significant effect on shoulder EMG activity regardless of patient population or muscle tested. Generally, the supraspinatus/upper trapezius muscle activity was significantly higher than the deltoid and infraspinatus activity—especially in the patients with pathological shoulders
Garofalo et al	2010	Effects of one-month continuous passive motion after arthroscopic rotator cuff repair: results at 1‑year follow-up of a prospective randomized study	RCT 2b/1−	100 54/46	Pain with the VAS scale (0–10) and the range of motion (ROM)	Our findings show that postoperative treatment of an arthroscopic rotator cuff repair with passive self-assisted exercises associated with 2‑h CPM a day provides a significant advantage in terms of ROM improvement and pain relief when compared to passive self-assisted exercise alone, at the short-term follow-up. No significant differences between the two groups were observed at 1 year postoperatively
Holmgren et al	2012	Supervised strengthening exercises versus home-based movement exercises after arthroscopic acromioplasty: a randomized clinical trial	RCT 2b/1−	36 18/18	Function, pain (Constant-Murley; CM) and Disabilities of the Arm, Shoulder, and Hand (DASH) scores, and health-related quality of life	The PT-group exhibited significantly greater improvements in CM (*p* = 0.02) and DASH (*p* = 0.05) scores. After treatment, the between-group mean difference in CM scores was 14.2 *p* (95% confidence interval 2 to 26). At the 6‑month follow-up, the between-group mean difference in DASH scores was 13.4 *p* (95% confidence interval 0.1 to 23)
Hultenheim Klintberg et al	2008	Early activation or a more protective regime after arthroscopic subacromial decompression—a description of clinical changes with two different physiotherapy treatment protocols—a prospective, randomized pilot study with a two-year follow-up	RCT 2b/1−	34 20/14	Pain, patient satisfaction, active range of motion and muscular strength were evaluated. Shoulder function was evaluated using Constant score, Hand in neck, Pour out of a pot and Functional Index of the Shoulder	Both groups showed significant improvements in pain during activity and at rest, in range of motion in extension and abduction, in strength of external rotation and in function. There were no clinical differences in changes between groups. Most patients were pain-free from six months. After two years, the majority of patients achieved ≥160 degrees in flexion, ≥175 degrees in abduction and 80 degrees in external rotation, the traditional achieved 67 and the progressive group 87 with Constant score
Keener et al	2014	Rehabilitation following arthroscopic rotator cuff repair: a prospective randomized trial of immobilization compared with early motion	RCT 2b/1−	124 67/62	Visual analog pain scale score, American Shoulder and Elbow Surgeons (ASES) score, Simple Shoulder Test (SST), relative Constant score, and strength measurements at six, twelve, and twenty-four months	There were no significant differences in patient age, tear size, or measures of preoperative function between groups at baseline. Final clinical follow-up was available for 114 subjects (92%). Active elevation and external rotation were better in the traditional rehabilitation group at three months. No significant differences were seen in functional scores, active motion, and shoulder strength between rehabilitation groups at later time points. Functional outcomes plateaued at six or twelve months except for the relative Constant score, which improved up to twenty-four months following surgery. Ninety-two percent of the tears were healed, with no difference between rehabilitation protocols (*p* = 0.46)
Kim et al	2012	Extracorporeal shock wave therapy is not useful after arthroscopic rotator cuff repair	RCT 2b/1−	71 35/36	Pain score (VAS), Constant score, University of California, Los Angeles (UCLA) score, ROM, manual muscle tes (MMT)	All patients were available for a minimum one-year follow-up. The mean age of the ESWT and control groups was 59.4 (SD: 7.7) and 58.6 years (SD: 7.8; n. s.). There were no significant differences in tear size and repair method between the two groups (n. s.). The mean Constant and UCLA scores, respectively, increased from 54.6 to 90.6 (*P* < 0.001) and from 18.5 to 27.4 (*P* < 0.001) in the ESWT group, and from 58.9 to 89.3 (*P* < 0.001) and 18.5 to 27.4 in the control group. Computed tomographic arthrography was performed in 26 patients from the ESWT group and 24 from the control group, and cuff integrity was maintained in 46 out of 50 patients. Definite re-tear was observed in two patients of the ESWT group and four of the controls. There were no complications associated with SWT
Kim et al	2012	Is early passive motion exercise necessary after arthroscopic rotator cuff repair?	RCT 2b/1−	117 60/57	Range of motion (ROM) and visual analog scale (VAS) for pain were measured preoperatively and 3, 6, and 12 months postoperatively. Functional evaluations, including Constant score, Simple Shoulder Test (SST), and American Shoulder and Elbow Surgeons (ASES) score, were also evaluated at 6 and 12 months postoperatively. Ultrasonography, magnetic resonance imaging, or computed tomography arthrography was utilized to evaluate cuff healing	There were no statistical differences between the 2 groups in ROM or VAS for pain at each time point. Functional evaluations were not statistically different between the 2 groups either. The final functional scores assessed at 12 months for groups 1 and 2 were as follows: Constant score, 69.81 ± 3.43 versus 69.83 ± 6.24 (*p* = 0.854); SST, 9.00 ± 2.12 versus 9.00 ± 2.59 (*p* = 0.631); and ASES score, 73.29 ± 18.48 versus 82.90 ± 12.35 (*p* = 0.216). Detachment of the repaired cuff was identified in 12% of group 1 and 18% of group 2 (*p* = 0.429)
Krischak et al	2013	A prospective randomized controlled trial comparing occupational therapy with home-based exercises in conservative treatment of rotator cuff tears	RCT 2b/1−	43 23/20	Pain intensity (VAS)	Two-thirds of the patients improved in clinical shoulder tests, regardless of the therapy group. There were no significant differences between the groups with reference to pain, range of motion, maximum peak force (abduction, external rotation), the Constant-Murley score, and the EQ-5D index. The only significant difference observed was the improvement in the self-assessed health-related quality of life (EQ-5D VAS) favoring home-based exercises
Lee et al	2012	Effect of two rehabilitation protocols on range of motion and healing rates after arthroscopic rotator cuff repair: aggressive versus limited early passive exercises	RCT 2b/1−	85 43/42	A postoperative MRI scan was performed at a mean of 7.6 months (range, 6 to 12 months) after surgery, strength, ROM	Regarding range of motion, group A improved more rapidly in forward flexion, external rotation at the side, internal and external rotation at 90degrees of abduction, and abduction than group B until 3 months postoperatively with significant differences. However, there were no statistically significant differences between the 2 groups at 1‑year follow-up (*p* = 0.827 for forward flexion, *p* = 0.132 for external rotation at the side, *p* = 0.661 for external rotation at 90degrees of abduction, and *p* = 0.252 for abduction), except in internal rotation at 90degrees of abduction (*p* = 0.021). In assessing the repair integrity with postoperative MRI scans, 7 of 30 cases (23.3%) in group A and 3 of 34 cases (8.8%) in group B had retears, but the difference was not statistically significant (*p* = 0.106)
Lisinski et al	2012	Supervised versus uncontrolled rehabilitation of patients after rotator cuff repair-clinical and neurophysiological comparative study	RCT 2b/1−	22 11/11	Pain level (visual analog scale), active range of motion (gonio-meter), activity of muscle’s motor units at rest and during maximal effort with electromyography and transmission of motor fibers in brachial plexus with electroneurography (M-wave stimulation studies)	In the group of supervised patients the active range of movement changed significantly from 26.4º to 101.5º on average for flexion with adduction while flexion with abduction improved from 21º to 95.5º. Pain sensation changed from 6.4 to 3.2. The mean resting electromyogram amplitude decreased to the greatest degree from 80.9 µV to 36.8 µV in trapezius muscle while maximal effort electromyogram amplitude increased in this muscle from 381.8 µV to 790.9 µV. The mean values of amplitudes in electroneurographical suprascapular nerve examinations increased from 536.4µV to 1691µV. No significant differences at *P* = 0.05 were found in these parameters recorded in the patients performing uncontrolled exercises
Long et al	2010	Activation of the shoulder musculature during pendulum exercises and light activities	Case-control study 3a/2−	17	Muscle activity (EMG)	Incorrect and correct large pendulums and drinking elicited more than 15% maximum voluntary isometric contraction in the supraspinatus and infraspinatus. The supraspinatus EMG signal amplitude was greater during large, incorrectly performed pendulums than during those performed correctly. Both correct and incorrect large pendulums resulted in statistically higher muscle activity in the supraspinatus than the small pendulums
Oh et al	2011	Effectiveness of subacromial anti-adhesive agent injection after arthroscopic rotator cuff repair: prospective randomized comparison study	RCT 2b/1−	80 40/40	Pain, passive range of motion (2, 6 weeks, 3, 6, 12 months after surgery), and the functional scores (6, 12 months postoperatively)	The HA/CMC injection group showed faster recovery of forward flexion at 2 weeks postoperatively than the control group but the difference was not statistically significant (*p* = 0.09). There were no significant difference in pain VAS, internal rotation, external rotation and functional scores between two groups at each follow-up period. The functional scores improved 6 months after surgery in both groups but there were no differences between the two groups. The incidence of unhealed rotator cuff was similar in the two groups. There were no complications related to an injection of anti-adhesive agents including wound problems or infections

In order to do justice to the amount of information contained in each publication, the individual sub-topics of the overall rehabilitation process are thematically assessed and discussed in individual sub-sections below. Furthermore, the results of the expert survey are presented according to topic.

## Immobilization and arm positioning

Directly after the operation, the question arises as to whether and to what extent the shoulder should be immobilized. The risk of a re-rupture or disrupted tendon healing as a result of too much strain have to be weighed against a stiff shoulder caused by too little mobilization. Cadaver studies reveal that the so-called “time zero strength” of the sutured supraspinatus tendon resists 70–100% of the forces affecting it [40]. However, biomechanical studies have shown there is a “gapping effect” for cyclical, clinically relevant strain, even in the case of double row suture techniques [40]. As tendon healing progresses, the biomechanical properties of the tendon-suture-construct change. Therefore, the time it takes for tendons to heal should be taken into account. Animal studies are frequently referred to, since the tendon healing process has already been intensively studied in animals. In animal models a fragile scar appears 0–14 days after the operation during the inflammatory phase [7]. In the subsequent proliferative phase, 3–4 weeks after the operation, fibroblasts, myofibroblasts and endothelial cells appear, neoangiogenesis begins, and a stronger tendon-bone connection develops. In the maturation and remodeling phase, starting in weeks 4 to 6, collagen III is increasingly replaced by mature collagen I and the tendon integrates more strongly and stably into the bone.

Animal studies have shown that the time it takes to achieve full strength varies between 12 and 26 months [7]. When the issue of early exercise therapy is translated to animal models, difficulties arise in comparing and interpreting the different animal models. It is also difficult to standardize any exercises for animals. Transferring the findings to humans also poses a challenge.

Li et al. [30] found that early passive exercise benefited tendon healing in rabbits. Peltz et al. [36] demonstrated in a rat model that movement was poorer when there was passive exercise directly after the operation as a result of increased scar formation. There were no differences with respect to tendon healing. By contrast, Gimbel et al. [16] found in rat models that the healing tendon had better mechanical properties when immobilization was extended. However, it is interesting to note that complete strain reduction using a botulinum toxin appears to have negative effects on tendon recovery in animal models [13]. In a comparison study of rabbits that compared immediately allowing movement, short-term immobilization with subsequent passive exercise and complete immobilization [47] Zhang et al. found that direct, post-operative passive exercise with intermittent immobilization did not negatively affect tendon healing histologically and in magnetic resonance imaging (MRI). However, tendon healing was found to diminish when function was completely allowed.

Compared to these heterogeneous animal studies, prospective studies of humans provide a good level of data. Early passive exercise does not appear to be disadvantageous [19]. Both Chan et al. [6] and Shen et al. [41] were able to show in meta analyses of randomized clinical comparative studies that no significant differences can be expected in the clinical outcome and in terms of the re-rupture rate. When there is early passive exercise, the full range of motion (ROM) is also achieved more quickly, particularly in terms of flexion. In a detailed evaluation of the meta analyses and our own additional review of the literature, a total of four Level I studies were identified that support the recommendation of early passive mobilization [2, 8, 20, 22].

By contrast, early aggressive active exercise should be avoided since this negatively impacts the healing process [20]. In order to protect patients from excessive strain outside the therapy setting, an aid can be used to immobilize the arm. Based on the timeframe of tendon healing mentioned above, the length of immobilization varies widely between 4 and 8 weeks [2, 4, 14, 21, 22, 29]. There are no prospective studies that deal only with the length of immobilization.

While the duration of immobilization is the subject of debate, immobilization in slight abduction is predominantly preferred by the experts surveyed as this increases blood circulation in the tendon and reduces the strain on the reconstruction [38]. Gerber et al. [15] and Thomopoulos et al. [45] were also able to show in animal models that a position that lowers the strain on the tendon reconstruction has a positive effect on the orientation of the collagen fibers and the elasticity of the tendon. Orthoses are, in principle, suitable for lowering the activity of the RC muscles. This was proven by Alenabi et al. [1] in an electromyographic study. When the elbow and hand were moved in a splint, the activity of the RC muscle was measured at no more than 10% of normal activity. There are no clinical investigations that specifically look at the type of orthoses used. The German catalog of medical aids allows both the use of arm slings and abduction pillows with a varying abduction of 15–45° for post-treatment after an RCR.

### Conclusions

Early passive, postoperative exercise can be used without indicating an increased rate of disruption of the healing process or ruptures. Employing an orthosis can protect against active strain that is applied too early. There are no evidence-based recommendations regarding the length of time that postoperative immobilization should last. The use of an arm abduction pillow can be considered (see Table 5 for DVSE expert opinions on immobilization).

**Table 5:** DVSE experts survey—Immobilization and arm positioning (no. of responds *n* = 44)

	Appropriate (%)	Rather appropriate (%)	Rather not appropriate (%)	Not appropriate (%)
Question 1: **After a RCR, the operated shoulder should be immobilized for 4–6 weeks, i.** **e. neither treated passively nor actively.** I consider this statement to be:	9.1	9.1	11.4	**70.5**
Question 2: **I think early** ^**a**^ ** passive exercise of the shoulder after RCR is beneficial.** I consider this statement to be:	**63.6**	22.7	9.1	4.5
Question 3: **I fear a relevant stiffening of the shoulder, if it is completely** ^**b**^ ** immobilized for the first 4–6 weeks after RCR.** I consider this statement to be:	**38.6**	34.1	22.7	4.5
Question 4: **I am afraid of a re-rupture or failure of tendon healing, if passive exercise starts at the first post-operative day after RCR.** I consider this statement to be:	6.8	13.6	34.1	**45.5**
	**No device**	**Sling**	**Brace (Abd:15–20°)**	**Brace (Abd:>20°)**
Question 5: **Do you recommend any kind of orthopedic orthosis, brace or sling after RCR, and if so, which one?**	2.3	27.9	**69.8**	11.6
Question 6: **What is the timeframe an orthosis/brace/sling should be worn?**	Min.: 1w–Max.: 12w; Ø: 4.9w; Median: 6w			

*Abd* abduction

^a^starting in the first post-operative week; ^b^no passive or active therapy peformed

## Physical therapy

Cryotherapy, electrotherapy and exercise in an exercise pool are frequent methods of physical therapy that are used following an RCR. In a randomized clinical trial (RCT) with 50 patients conducted in 1996, Speer et al. [43] investigated the effects of using cryotherapy systems after a variety of shoulder operations, including RCR. Continuous cryotherapy leads to a reduction in pain, a reduced need for pain killers and better sleep quality in the night after the operation. When cryotherapy was used (4 to 6 times per day depending on patient requirements) there was less pain when the arm was at rest and in motion in the 10 days following the operation. In another RCT, the same working group also observed clinically relevant effects on pain in the cryotherapy group when at rest and when physical strain was placed on the shoulder following open and arthroscopic shoulder operations (*n* = 70; water temperature 7–13 °C; length it was worn: continuously 48 h postoperatively; at night on days 3–7; daily 2–4 h on days 8–21 followed by exercise therapy; [42]). Speer’s group also demonstrated that continuous cryotherapy directly following reconstruction of the RC reduced the temperature in the glenohumeral joint and subacromial space by around 0.5–1.0 °C [35].

Blum et al. [4] compared two types of electrotherapy in an RCT with 22 patients who received RC reconstruction. The control group received 2 × 1 h of electrotherapy per day in connection with physiotherapy that started 6–8 weeks post-op. The intervention group received the same length of sham electrotherapy and physiotherapy that began 8 weeks after the operation.

In contrast to the control group, movement improved in the intervention group by around 10° 45 and 90 days after the operation, however strength did not. The methodological quality of the study should be regarded critically as the authors had a relevant conflict of interest.

A non-randomized study indicates that additional group sessions of aquatic theraphy (starting 10 days after reconstruction) have a positive effect on passive movement (anteversion and external rotation), pain and activities of daily living (Western Ontario Rotator Cuff Score) 3 and 6 weeks, though not 12 weeks, post operation [5]. However, the effects were slight and could also be the result of an overall higher amount of active intervention in the aquatic therapy group.

### Conclusions

Cryotherapy is recommended in the first 3 weeks following RCR in order to support rehabilitation and, in particular, to treat pain [43]. Based on current published studies, no clear recommendation can be made for or against electrotherapy, aquatic therapy, the application of heat, massages, therapeutic ultrasound, extracorporeal shockwave therapy and injections of hyaluronic acid [4, 5, 21, 34].

Individual studies indicate a potential benefit of electrotherapy and group training in an exercise pool (see Table 6 for DVSE expert opinions on physical therapy).

**Table 6:** DVSE experts survey—Physical therapy (no. of responds *n* = 44)

	Appropriate (%)	Rather appropriate (%)	Rather not appropriate (%)	Not appropriate (%)
Question 1: **The use of cryotherapy to reduce pain after a RCR is reasonable.** I consider this statement to be:	36.4	**40.9**	18.2	4.5
Question 2: **Electrotherapy plays a relevant role in the post-operative treatment after RCR.** I consider this statement to be:	6.8	13.6	**40.9**	38.6
Question 3: **Assisted active exercises as part of aquatic therapy (e.** **g. in a training pool) can improve active mobility after a RCR.** I consider this statement to be:	**45.5**	36.4	15.9	2.3

## Continuous passive motion

Continuous passive motion therapy with a motorized CPM machine is one of the most frequently used elements of treatment following an operation on a shoulder joint, and particularly after RCR. The passive motion machine typically serves to mobilize the joint shortly after the operation without the patient having to actively support the extension of motion.

Currently scientific literature only contains two reviews [3, 10] and one prospective randomized study [14]. The review by Baumgarten et al. [3] is based on two studies in which a CPM machine was used on 26 patients [37] and 31 patients respectively [28]. Both investigations compared physical therapy treatment (manual passive exercise) to the use of a CPM machine. Baumgarten et al. [3] concluded that the validity of the data is limited due to its poor methodological quality and provides insufficient evidence for the development of an evidence-based rehabilitation protocol. CPM treatment was not found to be superior. Du Plessis et al. [10] compared the effects of standard physical therapy to CPM therapy combined with physical therapy. The group of patients receiving CPM combined with physical therapy treatment, were given passive, isometric and actively supported exercises, shoulder mobilization and strength training. Manual passive mobilization, active exercise, and therapist coordinated self-exercise were used in the group that received standard physical training. Data on the range of movement, muscle strength, and pain reduction was collected, and studies previously conducted by Raab et al. [37] and Lastayo et al. [28], as well as a paper by Michael et al. [33] were also included. The authors of the review concluded that the use of a CPM machine in combination with physical therapy as part of follow-up treatment after an RCR can be regarded as safe [10]. A paper by Garofalo et al. [14] looked at 100 patients, comparing a standard program of passive exercise (therapist coordinated self-exercise: three series with 10 repetitions, pendulum movement, passive abduction, flexion and external rotation) to the same program with the addition of a CPM chair. This was applied for two hours a day for 4 × 30 min. In this comparison, the additional use of a CPM chair led to an improvement in results. It remains unclear, however, whether the device itself or the additional movement produced this effect [14].

### Conclusions

Based on these studies, no recommendation can be made with a high level of evidence for or against the use of CMP therapy following RCR, and not for the length of time, frequency and intensity of the CPM treatment. It should be noted, however, that passive motion exercise does not negatively impact the healing process (see Table 7 for the DVSE expert opinions on CPM).

**Table 7:** DVSE experts survey—Physiotherapy, self/home-exercises and continuous passive motion (no. of responds *n* = 44)

	Appropriate (%)	Rather appropriate (%)	Rather not appropriate (%)	Not appropriate (%)	
Question 1: **The use of self/home-exercises makes sense in the early** ^**a**^ ** post-operative phase after RCR.** I consider this statement to be:	**36.4**	20.5	22.7	20.5	
Question 2: **I hand out a post-operative exercise plan to the patient.** I consider this statement to be:	**38.6**	18.2	9.1	34.1	
Question 3: **The initial instruction of self/home-exercises after a RCR by a physiotherapist makes sense.** I consider this statement to be:	**68.2**	27.3	4.5	0	
Question 4: **The visualization of self/home-exercises (by photo/video) makes sense.** I consider this statement to be:	**59.1**	29.5	6.8	4.5	
Question 5: **Self/home-exercises supersede physiotherapy units after RCR** **.** I consider this statement to be:	9.1	11.4	**40.9**	38.6	
Question 6: **The use of a continuous passive motion (CPM) device makes sense during the post-operative treatment after RCR.** I consider this statement to be:	22.7	13.6	**34.1**	29.5	
	No CPM	1 × 30min^b^	2 × 30min^b^	3 × 30min^b^	4 × 30min^b^
Question 7: **What is the frequency CPM therapy should be performed?**	**56.8**	4.5	18.2	13.6	6.8

^a^starting in the first post-operative week; ^b^per day

## Self-exercise

In addition to CPM, self-exercise is another important component of post-operative follow-up treatment which is used to varying degrees [8, 11, 18, 29]. There are major differences in point in time, intensity, type of exercise and supporting measures. Patients can be instructed through written directions, videos and/or receive instruction from the physiotherapist (PT). Roddey et al. [39] found there was no significant difference in the post-operative outcome when the instructions were given by the PT or by video.

Pendulum exercises were often described in the first post-operative phase. Biomechanical studies have shown that it is important to carry them out correctly so that there is low electromyographic activity (EMG) in the reconstructed RC (see below, [32]). The lowest activity was recorded in small pendulum circles (d = 20 cm) with an initiation of the arm movement by moving the torso and not by using the shoulder muscles themselves [32]. Additional use of a 1.5 kg weight on the hanging arm increases the EMG activity of M. supraspinatus and M. infraspinatus, though not to a statistically significant degree [12]. Furthermore, mobilization exercises (with the help of the contra-lateral arm) and, in later phases, muscle activation/strengthening exercises using simple devices (e. g. theraband, dumbbells) are other primary forms of self-exercise [8, 11, 46]. There is no homogeneous data on the extent of the passive mobilization and when to start it [2, 8, 21, 29]. The question of whether self-exercise, in addition to physiotherapy, has a positive effect cannot be sufficiently answered. Both are combined in many published studies, however a direct comparative study currently does not exist [1, 11, 31, 33, 46]. Scientific literature contains only two randomized controlled trials that look at self-exercise versus physiotherapy exercise [3]. In their Level II study, Hayes et al. [17] were able to randomize 58 patients into two control groups. After both groups received instructions on the self-exercise program in the first week, one group subsequently received physiotherapy treatment while the other group continued to do the self-exercise program. No significant differences in ROM, strength measurement and shoulder scores were found at any of the follow-up treatments (6, 12 and 24 weeks). Critical aspects of the study include the low number of cases, the high conversion rate from the self-exercise group to the physiotherapy group (*n* = 9) and the high drop-out rate (27%).

The second study by Lee et al. conducted a clinical and neurophysiological examination of 11 patients per group at days 20 and 40 post operation [29]. The results showed significantly better active mobility at both follow-ups in the group receiving physiotherapy. This group also achieved a significant improvement in the activation of motor units in the EMG; this was not detected in the self-exercise group. In this study the low number of cases, the short follow-up period and an absence of clinical scores should be viewed critically.

### Conclusions

No Level I-based recommendation for or against the use of self-exercise can be made, however, based on the available studies, its use can be considered (see Table 7 for DVSE expert opinions on self-exercise).

## Physiotherapy and the phase model

### Rehabilitation phases/protocols (time- and criteria-based)

In order to enable continuous progression of rehab-treatment, the post-operative process should be divided into different phases. The available literature usually breaks the process down into four phases. Thus, the different treatment focuses and the corresponding targets can be usefully classified [23, 27, 40, 44].

- The first phase is the time directly after the operation until week 6. During this time mainly passive and assistive exercises are conducted.
- This is followed by Phase 2 that lasts a further 6 weeks during which active functions are regained (week 7–12 post operation).
- Phase 3, strength building, starts in the third post-operative month (month 3 and 4).
- This is concluded by Phase 4 which includes the return to sports (Table 8; [20]).

**Table 8:** Four-phase model/protocol of rehabilitation

Phase and duration	Targets according to ICF	Contents	Milestones before transition to next phase	ADL and core exercises
I: Day 1 after surgery up to week 6 [20]	**Body functions:** – Reducing pain, facilitating resorption – Preserving/improving joint mobility – Regulating affected vegetative and neuromuscular functions – Improving joint stability – Tendon healing and preventing post-operative adhesions – Preventing structural damage – Improving the functions affecting the sensory motor system – Learning the optimal positioning of the scapula and centering of the humeral head	– Immobilization (as a form of protection) in 15–45° ABD – ABD orthosis/sling/brace can be removed during showers, while eating and for physiotherapy [8] – Pendulum exercise [8, 26, 46] – Aquatic therapy if wounds are intact [5] – CPM if favored [10] – No active shoulder joint movement against resistance – Limitation: 30° ER, flex and ABD 90° in a pain-free range [29, 37], avoid ADD PROM – Assistive active exercise in a pain-free range can begin in week 4, taking into account the ROM limitation [5, 33, 46]	Symmetrical and pain-free movement compared to opposite side: – PROM flexion 90° – PROM ER and IR with adjacent scapula 45° – PROM ABD with adjacent scapula 90°	– Pendulum exercise in elevation [8, 26, 46] – Elev. in closed chain: stand in front of the table and stretch out arms – Active movement of elbow, wrist and fingers [46] – Keeping posture erect and controlling scapula [26] – Isolated scapula depression and protraction [46] – At the end of the phase: aqua training [46]
	**Activities/participation:** – Going about daily routine while alleviating the arm that has been operated on – Facilitating mobility – Breaking down barriers that make ADLs difficult			
II: Week 6–12 [20]	**Body functions:** – Tissue healing, full PROM, developing dynamic shoulder stabilization, reducing pain, reducing inflammation [3] – Tendon healing and remodeling phase “low level loading” is permitted – Scar mobilization to prevent adhesions – Promoting resorption – Improving functions affecting the sensory motor system – Regulating affected vegetative and neuromuscular functions – Improving the functions of muscle strength – Preventing structural damage – Full AAROM transitioning to AROM against force of gravity – Improved kinematics of the shoulder joint and scapula setting [9]	– Full AAROM transitioning to AROM against force of gravity – Scar mobilization – Aqua gymnastics/aquatic therapy [5] – CPM if favored [10] – Training in closed chain to build up strength – Training in open chain to improve intramuscular coordination – Limitation: up to the pain threshold [8] – No resistance or strengthening exercises	– Active achievement of all possible active range of movements [9] – No scapulothoracic dysfunction – Sufficient glenohumeral and scapulothoracic functionality [9]	– Back position: support affected side with non-affected side and move arm above the head (AAROM; [46]) – Training of everyday movements—eating, combing hair, getting dressed etc [46] – Stabilization in closed chain – Proprioceptive training in an open chain [46] – Isometric strengthening of RC to a max. of 50% of strength
	**Activities/participation:** – Carrying out daily routine (household, personal hygiene) – Correcting posture (developing ergonomic posture) – Mobility (carrying/lifting objects, using arm-hand) – Participating in social activities – Following an independent home training program			
III: Month 3–4 [20]	**Body functions:** – Full AROM – Dynamic shoulder stabilization, regaining strength and flexibility, regaining functional activities – Improved kinematics of the shoulder joint [9] – Participating in work and social life [9] – Improving the functions affecting the sensory motor system	– Building up strength—slowly starting to build up strength—low level [8] – Stretching – Avoiding overhead exercises	– Free functional movement in a pain free range [9] – ADL possible without pain—avoiding overhead exercises – If enough strength in RC, Phase 4 can start in order to carry out ADL cleanly and without pain – 75% of normal strength and endurance [9]	– Light functional exercises [26] – Mobilization/building up strength using a rope pull with low weights [26, 46] – Push-ups against the wall [11] – Bicep and tricep training with low free weights [46]
	**Activities/participation:** – Developing an ergonomic posture in daily life/at work/during sports – Mobility – Regaining trust in movement and shoulder stability – Return to work – Participating in social activities – Following an independent home training program			
IV: Month 4–6 [20]	**Body functions:** – Achieving full and pain-free AROM, improving strength and flexibility, redeveloping functional activities	– Stretching – Strengthening functional training	– Return to sports after 6 months if [46]: – Mobility and strength are symmetrical with the opposite side – Normal scapulothoracic movement is present – There is no pain at rest and during activity	– PNF against resistance [26, 46] – Explosive strength training [9] – Training in a specific sport in the pain-free range [9]
	**Activities/participation:** – Regaining kinematics related to sports, daily life and work [9] – Improving endurance and explosive strength [9]			

^a^No participation in overhead sports until all muscular deficits in the shoulder girdle are settled and patient is free of pain [9]; no overhead and contact sports until 6 months post-op and only after consulting a physician [46]

*AAROM* assistive-active range of motion, *AROM* active range of motion, *ABD* abduction, *ADD* Adduction, *CPM* continuous passive motion, *ER* external rotation, *ADL* activity of daily living, *Elev* elevation, *Flex* Flexion, *IR* internal rotation, *PNF* proprioceptive neuromuscular facilitation, *PROM* passive range of motion

The timeline is aligned with the general phases of wound healing and the time it takes for tissue to heal, as identified in animal studies. These time markers define the framework for the follow-up treatment phases. There is a consensus that rehabilitation should be improved both in terms of time and criteria [9]. The literature defines no precise criteria that should act as the specific criteria which the patient should fulfil before moving on to the next rehabilitation phase. However, the “International Classification of Functioning, Disability and Health” (ICF) is a good basis for identifying targets. Orientational criteria are assigned to each phase as listed in Table 8.

The four-phase model is structured as follows: Phase 1 lasts 6 weeks starting on the first day after the operation [20]. During this time, the key targets include achieving good tendon healing without post-operative adhesions and, above all, reducing pain for the patient. Throughout Phase 1 the shoulder should be immobilized in a position at15–45° abduction using an orthosis/sling/brace which is only taken off during physiotherapy and for hygiene purposes [8]. Only a passive exercise program is allowed until the end of the fourth week after the operation. Then, depending on the amount of pain the patient is still in, assistive exercise can be integrated into the treatment program [5, 33, 46]. Nevertheless, the extent of motion is limited to 30° external rotation, 90° flexion and abduction in a pain-free range [29, 37]. Adduction should be avoided both in a passive and assistive fashion. The core exercises in this phase are pendulum and scapula-thoracic exercises. All of the exercises to passively expand elevation are only allowed in a closed chain [1]. Only the exercising of the adjacent joints, elbow, hand and fingers is active and allowed in an open chain [1, 26, 46]. After 6 weeks and at the end of Phase 1, a passive flexion up to 90°, a passive internal and external rotation with adjacent scapula up to 45° and a passive abduction, also with an adjacent scapula up to 90° on the operated side, should be possible. The movement should be symmetrical to the opposite side and pain free.

The activities of Phase 2 are done until week 12 following the operation. The goals of this phase include tissue healing, achieving a full passive range of movement and the development of dynamic shoulder stabilization. In this phase of tendon healing and remodeling, only “low level loading” is allowed. At the same time, scar mobilization is an important element to prevent adhesions. By the end of this phase, the full range of movement can be trained in an active-assistive fashion and an active increase in movement against the force of gravity can start. This targets the improvement of the kinematics of the shoulder joint [9].

Twelve weeks after the operation the patient should also actively achieve the degree of motion that was achieved passively up to this point in time. It should be noted that by now there should no longer be any scapulothoracic dysfunction [9]. Once there is sufficient glenohumeral and scapulothoracic movement, the therapy can move on to Phase 3 [9].

In Phase 3 (month 3 and 4 post operation) the full active range of movement and dynamic shoulder stabilization should be achieved. At this point in time, tendon healing should have progressed enough to integrate strengthening and stretching as additional elements in this phase so that patients can regain functional activity and participate in their professional and social lives [9]. Light functional exercises and mobilization/strengthening exercises using a pulley with low weights are a good way to do this at this time [26, 46]. Push-ups against the wall [5, 11] and bicep and tricep exercises with low free weights or a resistance band are once again permitted [46].

At the end of post-operative month 4 the patient should have regained full functional movement within the pain-free range and be able to perform activities of daily living (ADL) without pain [9]. At this point in time, around 75% of normal strength and endurance has been reestablished [9]. When there is sufficient strength in the RC to carry out ADL cleanly and without pain, Phase 4 can start. In the fourth and final phase, which extends up to 6 months after the operation, training focuses on maintaining a final and pain-free active range of movement, improving strength and flexibility, and improving endurance and explosive power [9]. Regaining functional activities and reestablishing kinematics related to sports, daily life and work are the aims of this final phase as defined by the ICF [9]. A return to sports isn’t permitted until the end of this final phase and until mobility and strength are symmetrical with the opposite side. Further requirements are normal scapulothoracic mobility and no pain at rest and during activity ([46]; see Table 7 and 9 for DVSE expert opinions on physiotherapy and the phase model).

**Table 9:** DVSE experts survey—Rehabilitation protocol (no. of responds *n* = 44)

	Appropriate (%)	Rather appropriate (%)	Rather not appropriate (%)	Not appropriate (%)
Question 1: **The rehabilitation protocol after RCR should has a progressive exercise set-up and can be divided into 4 phases.** I consider this statement to be:	**63.6**	34.1	2.3	0
Question 2: **The phase transitions and load increases should be time-based and criteria-based.** I consider this statement to be:	**81.8**	18.2	0	0

## Conclusions for clinical practice

Today, RCR is an established standard procedure. The post-operative follow-up treatment period is expected to be long and time-consuming. The contents and concepts of therapy are, thus, applied in different ways and controversially discussed. The number of publications on the subject is therefore high. Unfortunately, not all of the papers fulfil the required quality criteria of evidence-based medicine.

Since 2004 one guideline, four reviews and 17 original papers have been identified that serve as the basis for establishing structured follow-up treatment. For some treatments, clear recommendations can be derived. These include early passive exercise, using cryotherapy to reduce the pain, self-exercise and the use of orthoses. Despite this, there are still questions that cannot be answered conclusively based on the available literature. When all of the results were looked at together, a basic concept that was solid and valid could nevertheless be created which was summarized in a four-phase model. The main points of this model were supported and supplemented for the first time through collected and pooled expert opinions from the DVSE expert society.
